# Association Between Persistent Maternal Depression among Japanese New Mothers and their Toddlers’ Behaviors

**DOI:** 10.1007/s10995-025-04049-y

**Published:** 2025-01-20

**Authors:** Haruka Tamura, Naoko Nishitani

**Affiliations:** Department of Nursing for Community-based Integrated Care, Division of Integrated Health Sciences, Nagoya University Graduate School of Medicine, 1-1-20 Daiko- Minami, Higashi-ku, Nagoya City, Aichi Prefecture Japan

**Keywords:** Persistent maternal depression, Mental health, Maternal lifestyles, Child development, Parenting

## Abstract

**Objectives:**

To determine the association between mothers’ persistent maternal depression and their toddlers’ behavior.

**Methods:**

Online surveys were conducted twice with mothers who gave birth to their first child between March and June 2020. The survey periods were November 2020 and May–June 2022. Measures included baseline characteristics and family environment factors, maternal postpartum depression (Edinburgh Postnatal Depression Scale [EPDS]), maternal lifestyle and mother-reported toddler behaviors, and Internet/media use. Statistical analysis was performed using the χ² test, trend test, and logistic regression.

**Results:**

Of the 339 participants, 82 (24.1%) were in the “persistent maternal depression” group with high EPDS scores (≥ 9 points) at both time points, and 178 (52.5%) were in the “no maternal depression” group with low EPDS scores (< 9 points) at both time points. Persistent maternal depression was associated with sleep, eating behavior, physical activity, parenting emotions, and abusive behavior of mothers. Furthermore, persistent maternal depression may be related to undesirable toddler behaviors such as smartphone overuse and hyperactivity-like symptoms.

**Conclusions for Practice:**

The persistence of postpartum depression is influenced by factors such as mothers’ parenting emotions. Preventing and supporting maternal depression in mothers can foster favorable behaviors in toddlers. In Japan, enhanced individualized, ongoing support for postpartum mothers, tailored in duration and form, could promote both maternal well-being and positive parenting practices.

## Significance

**What is Already Known on this Subject?:**

Toddler health in Japan is greatly affected by toddlers’ environment and family behaviors. Maternal depression, linked to lifestyle and social factors, impacts both mothers and toddlers, potentially causing behavioral issues.

**What does this Study Add?:**

Persistent maternal depression was associated with undesirable toddler behaviors. More tailor-made support options for maternal depression, differing in duration and form according to specific needs, are required; providing these options on an ongoing basis would also lead to desirable parenting behaviors. Preventing and providing support for maternal depression can promote healthy behavior in toddlers.

## Introduction

Toddlerhood is an important period of remarkable growth and development, both physically and mentally, during which the foundation of one’s personality is laid. Lifestyle behaviors such as eating, sleeping, exercising, and playing greatly influence development during this period. In Japan, the environment surrounding children has changed dramatically with the declining birth rate and the shift to nuclear families. Furthermore, the COVID-19 pandemic has highlighted new issues such as increased sedentary time, use of media devices, and other diversifying behaviors among children (López-Bueno et al., 2021). In particular, the COVID-19 pandemic was associated with an increased prevalence of overweight Japanese elementary school students in a study conducted in Tokyo, Japan, highlighting, among other things, a decrease in physical activity (Kawahara et al., 2024). These changes have been observed in all generations, including pregnant women, mothers, and toddlers, and have a negative impact on toddlers’ health-related behaviors, leading to worsening physical and mental health (Androutsos et al., 2021; López-Bueno et al., 2020, 2021).

The health of toddlers is greatly affected by their environment and family behavior, especially in the home. In Japan, the burden of housework and childcare is still disproportionately placed on mothers, although awareness of gender equality is progressing with an increase in the number of dual-earner households. According to a Japanese survey, mothers in Japan spend approximately 2.1 − 3.6 times as much time as fathers do on housework and childcare (Cabinet Office, 2020). Furthermore, multiple studies on the impact of mothers’ lifestyles on toddlers’ health have shown lifestyle transfers between parents and toddlers (Wang et al., 2022; Larsen et al., 2015). In general, maternal behaviors such as healthy eating and proper sleep habits play an important role in supporting mothers’ mental health and toddlers’ healthy growth.

The mother’s lifestyle, in addition to its impact on the toddler, is closely related to the mother’s own physical and mental health. Maternal depression is widely recognized as a common health problem faced by postpartum mothers worldwide and is attributed to lifestyle and environmental factors such as lack of sleep and social support, and can last for months or years (Lin et al., 2018; National Institutes of Health (NIH 2020). In the United Kingdom (Barker et al. 2012), Australia (Woolhouse et al., 2015), Canada (Wickham et al., 2015), and France (van der Waerden et al., 2015), studies have reported that 10–30% of mothers experience persistent maternal depression. Previous studies have shown that toddlers raised by mothers with persistent maternal depression experience emotional and behavioral difficulties (Giallo et al., 2015) and that persistent maternal depression is significantly associated with children’s psychological problems (Tainaka et al., 2022).

However, globally, there is limited research on how maternal depression specifically impacts toddler behavior, and further investigation in this area is needed. Therefore, the purpose of this study is to clarify the association between persistent maternal depression and toddler behavior in Japan. By elucidating the relationship between maternal mental health and infant development, this study provides a foundation for proposing specific measures to support the healthy development of toddlers.

## Methods

### Research Design and Duration

This was a longitudinal study with an online descriptive questionnaire survey administered in Japan. We conducted two surveys: the first from November 26 to 30, 2020 and the follow-up from May 26 to June 26, 2022.

### Sample Selection

Participants comprised mothers who gave birth to their first child between March and June 2020 and were registered respondents (1.3 million) of Macromill, Inc. (Tokyo, Japan), an Internet research company. The first screening randomly selected approximately 40,000 people who met the following eligibility criteria: (1) aged 18–44 years, (2) female, (3) married, and (4) with a child. For the second screening, eligibility criteria were based on raising a first child between 5 and 8 months postpartum and owning a smartphone. Participants were emailed the research outline, and were asked to review the eligibility and instructions and confirm their consent to participate. A total of 771 participants responded to the first survey. The follow-up survey was conducted one and a half years later and yielded 344 responses (44.6% response rate). Notably, this survey excluded mothers who gave birth to twins, did not give birth themselves (e.g., adoption), were not living with their toddler, and were unable to respond in Japanese.

### Measures

Measures assessed (1) basic attributes and family environment factors, (2) maternal depression, (3) lifestyles of mothers and mother-reported toddler behavior, and (4) Internet/media use. These variables were assessed in the second survey.

#### Basic Attributes and Family Environment

Participants were asked to respond to questions regarding age, employment status, family structure, daytime childrearing status, average daily hours of childrearing time, and rest time to ascertain their living conditions. Additionally, the participants were asked about their feelings regarding childrearing (whether they felt relaxed and confident in their childrearing) and abuse-suspect behaviors, such as hitting the child.

#### Maternal Depression

The Japanese version of the Edinburgh Postnatal Depression Scale (EPDS) was used to assess maternal depression in mothers (Cox et al., 1987; Okano, 1996). The EPDS has been used with mothers during pregnancy and several years postpartum (Netsi et al., 2018; Paul & Pearson, 2020). The Japanese version of the EPDS has adequate reliability with Cronbach’s α = 0.78, sensitivity of 0.75, and specificity of 0.93 when the cutoff is set at 9 points (Okano, 1996).

The EPDS was utilized in both the first and second surveys. Participants who scored < 9 in both surveys were defined as the “no maternal depression” group, and those who scored ≥ 9 in both surveys were defined as the “persistent maternal depression” group.

#### Lifestyles of Mothers and Mother-Reported Toddler Behavior

Based on previous studies, participants were asked about their own and their toddlers’ diets, physical activity, and sleep status (National Institute of Health and Nutrition, 2012; Ministry of Agriculture, Forestry and Fisheries, 2021; National Center for Child Health and Development, 2018). Some of these data were continuous, but were reclassified.

The Japanese version of the Athens Insomnia Scale (AIS) was administered to the participants. The AIS is a universal measure of insomnia developed by the Global Project on Sleep and Health, an initiative of the World Health Organization (Soldatos et al., 2000). Scores are quantified, with a maximum of 24 points to measure the degree of insomnia objectively. The AIS has been proven reliable and valid in healthy adults (Okajima et al., 2013).

Participants were also asked about their toddler’s health status and developmental indicators. Based on the project and a health checkup guide, we asked about the child’s progress at 18 months according to developmental milestones (National Center for Child Health and Development, 2018; Ministry of Health, Labour and Welfare, 2015) and history of health checkups. To obtain accurate results, participants were asked to refer to the Maternal and Child Health Handbook, which is distributed to all pregnant women in Japan and contains records of medical checkups and vaccinations by medical institutions and parents, as well as official records from medical institutions and government agencies.

#### Internet/Media Use

Participants were asked about their own and their child’s average daily hours of Internet/media use during the past week by type of device (smartphone, tablet, or TV). Participants were referred to the “weekly report function” on their smartphones to retrieve actual usage data. The “weekly report function” is a default feature on smartphones that records the time spent in operation. A simple operation shows, for example, the average screen time spent on the smartphone and each application over the previous week. External validity was reinforced by using this function and providing a supplementary explanation of how to operate it during data collection. We inquired about their usage of the Internet/media before going to bed.

## Analysis

### Participant Analysis

Of the 344 participants in the first and second surveys, excluding those who were not living with their toddlers, data from 339 participants (valid response rate: 98.5%) were included in the analysis.

### Statistical Analysis

The χ^²^ test and trend test (Mantel-Haenszel test) confirmed the association between persistent maternal depression and lifestyle. Furthermore, a logistic regression analysis was conducted with toddler’s behavior as the dependent variable, persistent postpartum depression as the independent variable, and age and employment as the adjusted variables. For all tests, *p* < 0.05 was considered statistically significant. SPSS Statistics version 27.0 for Windows (IBM Corp., Armonk, NY, USA) was used for the analysis.

## Results

Of the 339 participants, 82 (24.1%) were in the persistent maternal depression group with high EPDS scores (≥ 9 points) on both occasions, and 178 (52.5%) were in the no maternal depression group with low EPDS scores (< 9 points) on both occasions. The EPDS scores of the others (*n* = 79, 23.3%) fluctuated between being above and below 9 points. EPDS scores averaged 8.354 (standard deviation = 4.065) in the first survey and 6.558 (standard deviation = 4.968) in the second survey.

Persistent maternal depression was significantly different based on the mothers’ lifestyle elements of sleep (AIS, sleep duration), eating behavior (not eating three meals per day, adequate amount of food), physical activity (moderate to vigorous exercise), abuse-suspect behaviors, and parenting emotions (caring for toddler with a relaxed attitude, confidence in parenting; Table 1).

**Table 1:** Association between mothers’ lifestyles and presence of persistent postpartum depression in mothers

			Total(*n* = 260)		Persistent postpartum depression				
					Persistent postpartum depression group (*n* = 178)		No postpartum depression group (*n* = 82)		
Mothers’ lifestyles at 2 years old			*n*	%	*n*	%	*n*	%	*P* value
Basic attributes	Age	< 35 years	184	70.80%	127	71.30%	57	69.50%	0.762 a
		≥ 35 years	76	29.20%	51	28.70%	25	30.50%	
	Employment	Not working	121	46.50%	83	46.60%	38	46.30%	0.966 a
		Working	139	53.50%	95	53.40%	44	53.70%	
Sleep	AIS-J	< 3 points	98	37.10%	91	50.60%	7	8.30%	< 0.001 *a < 0.001 *b
		4 − 5 points	45	17.00%	31	17.20%	14	16.70%	
		≥ 6 points	121	45.80%	58	32.20%	63	75.00%	
	Sleep duration	< 5 h	38	14.60%	18	10.10%	20	24.40%	0.010 *a 0.010 *b
		5–8 h	199	76.50%	143	80.30%	56	68.30%	
		≥ 8 h	23	8.80%	17	9.60%	6	7.30%	
Meals	Three meals per day	Every day	183	70.40%	140	78.70%	43	52.40%	< 0.001 *a
		Not every day	77	29.60%	38	21.30%	39	47.60%	
	Adequate amount of food	Every day	157	60.40%	122	68.50%	35	42.70%	< 0.001 *a
		Not every day	103	39.60%	56	31.50%	47	57.30%	
	Eat a well-balanced diet	Every day	73	28.10%	50	28.10%	23	28.00%	0.995 a
		Not every day	187	71.90%	128	71.90%	59	72.00%	
	Eat slowly at least once a day	Every day	89	34.20%	62	34.80%	27	32.90%	0.764 a
		Not every day	171	65.80%	116	65.20%	55	67.10%	
	Take-out use	Less than once per week	79	30.40%	56	31.50%	23	28.00%	0.578 a
		More than once per week	181	69.60%	122	68.50%	59	72.00%	
Activity	Moderate to vigorous exercise habits	Having	46	17.70%	24	13.50%	22	26.80%	0.009 *a
		None	214	82.30%	154	86.50%	60	73.20%	
	Light physical activity habits	Having	145	55.80%	101	56.70%	44	53.70%	0.642 a
		None	115	44.20%	77	43.30%	38	46.30%	
Internet/media use	Duration of smartphone use	< 1 h	27	10.40%	18	10.10%	9	11.00%	0.832 a
		≥ 1 h	233	89.60%	160	89.90%	73	89.00%	
	Duration of tablet use	< 1 h	245	94.20%	171	96.10%	74	90.20%	0.061 a
		≥ 1 h	15	5.80%	7	3.90%	8	9.80%	
	Duration of TV use	< 1 h	122	46.90%	86	48.30%	36	43.90%	0.508 a
		≥ 1 h	138	53.10%	92	51.70%	46	56.10%	
Environment	Daily child care support	Present	36	13.80%	23	12.90%	13	15.90%	0.525 a
		None	224	86.20%	155	87.10%	69	84.10%	
	Parenting time by mother	< 1 h	2	0.80%	1	0.60%	1	1.20%	0.467 a 0.222 b
		1–2 h	16	6.20%	9	5.10%	7	8.50%	
		≥ 2 h	242	93.10%	168	94.40%	74	90.20%	
	Relaxing time by mother	< 1 h	111	42.70%	72	40.40%	39	47.60%	0.221 a 0.756 b
		1–2 h	86	33.10%	65	36.50%	21	25.60%	
		≥ 2 h	63	24.20%	41	23.00%	22	26.80%	
Parenting behavior	Experiencing abuse-suspect behaviors	Having	96	36.90%	47	26.40%	49	59.80%	< 0.001 *a
		None	164	63.10%	131	73.60%	33	40.20%	
Parenting emotion	Treating children with a relaxed attitude	Yes	189	72.70%	142	79.80%	47	57.30%	< 0.001 *a
		No	25	9.60%	8	4.50%	17	20.70%	
		Neutral	46	17.70%	28	15.70%	18	22.00%	
	No confidence in childcare	Yes	125	48.10%	70	39.30%	55	67.10%	< 0.001 *a
		No	75	28.80%	59	33.10%	16	19.50%	
		Neutral	60	23.10%	49	27.50%	11	13.40%	

*= *P* < 0.05, a = χ² test P, b = trend test P

AIS, Athens Insomnia Scale

Toddler behaviors associated with mothers’ persistent maternal depression were sleep (sleep duration and time to fall asleep), eating behaviors (not missing meals and eating slowly), indoor sitting time, smartphone use time, tablet use time, and hyperactivity-like symptoms (Table 2). A logistic regression analysis was conducted with toddler behavior as the dependent variable, persistent maternal depression as the independent variable, and age and employment as the adjusted variables. The results revealed that persistent maternal depression was associated with toddler spending more time on a smartphone/tablet (odds ratio [OR] = 1.757, 95% confidence interval [CI] = 1.319–2.340, and OR = 1.565, 95% CI = 1.123–2.179), device use before bedtime (OR = 1.316, 95% CI = 1.068–1.623), shorter sleep duration (OR = 1.699, 95% CI = 1.337–2.160), longer time to fall asleep (OR = 1.412, 95% CI = 1.133–1.760), not having three meals per day (OR = 1.458, 95% CI = 1.136–1.871), not eating slowly (OR = 1.251, 95% CI = 1.044–1.499), and hyperactivity-like symptoms (OR = 1.413, 95% CI = 1.176–1.697) (Table 3).

**Table 2:** Association between toddlers’ behaviors and presence of persistent postpartum depression in mothers

			Total(*n* = 260)		Persistent postpartum depression				
					Persistent postpartum depression group (*n* = 178)		No postpartum depression group (*n* = 82)		*P* value
Toddlers’ behaviors at 2 years old			*n*	%	*n*	%	*n*	%	
Sleep	Sleep duration	< 8 h	44	16.70%	17	9.40%	27	32.10%	< 0.001 *a <0.001 *b
		8–12 h	203	76.90%	152	84.40%	51	60.70%	
		≥ 12 h	17	6.40%	11	6.10%	6	7.10%	
	Time to fall asleep	< 1 h	215	82.70%	156	87.60%	59	72.00%	0.002 *a
		≥ 1 h	45	17.30%	22	12.40%	23	28.00%	
Meal	Three meals per day	Every day	227	87.30%	163	91.60%	64	78.00%	0.002 *a
		Not every day	33	12.70%	15	8.40%	18	22.00%	
	Adequate amount of food	Every day	165	63.50%	118	66.30%	47	57.30%	0.163 a
		Not every day	95	36.50%	60	33.70%	35	42.70%	
	Eats a well-balanced diet	Every day	105	40.40%	73	41.00%	32	39.00%	0.762 a
		Not every day	155	59.60%	105	59.00%	50	61.00%	
	Eats slowly at least once a day	Every day	161	61.90%	119	66.90%	42	51.20%	0.016 *a
		Not every day	99	38.10%	59	33.10%	40	48.80%	
	Drinks a sweet drink	Less than once per week	75	28.80%	57	32.00%	18	22.00%	0.096 a
		More than once per week	185	71.20%	121	68.00%	64	78.00%	
Physical activity	Outdoor playing time per day	< 30 min	67	25.40%	38	21.10%	29	34.50%	0.065 a 0.063 b
		30 min to 1 h	95	36.00%	69	38.30%	26	31.00%	
		≥ 1 h	102	38.60%	73	40.60%	29	34.50%	
	Indoor activity time per day	< 30 min	61	23.10%	37	20.60%	24	28.60%	0.273 a
		30 min to 1 h	79	29.90%	58	32.20%	21	25.00%	0.407 b
		≥ 1 h	124	47.00%	85	47.20%	39	46.40%	
	Indoor sitting time per day	< 30 min	56	21.20%	27	15.00%	29	34.50%	0.001 *a 0.004 *b
		30 min to 1 h	69	26.10%	52	28.90%	17	20.20%	
		≥ 1 h	139	52.70%	101	56.10%	38	45.20%	
Internet/media use	Duration of smartphone use	< 1 h	233	89.60%	169	94.90%	64	78.00%	< 0.001 *a
		≥ 1 h	27	10.40%	9	5.10%	18	22.00%	
	Duration of tablet use	< 1 h	242	93.10%	171	96.10%	71	86.60%	0.005 *a
		≥ 1 h	18	6.90%	7	3.90%	11	13.40%	
	Duration of TV use	< 1 h	112	43.10%	77	43.30%	35	42.70%	0.931 a
		≥ 1 h	148	56.90%	101	56.70%	47	57.30%	
	Device use before bedtime	Yes	50	19.20%	27	15.20%	23	28.00%	0.014 *a
		No	210	80.80%	151	84.80%	59	72.00%	
Development	Results of 18 months health checkup	No problem	252	96.90%	174	97.80%	78	95.10%	0.254 a
		Others	8	3.10%	4	2.20%	4	4.90%	
	Speaks meaningful words	No	15	5.80%	9	5.10%	6	7.30%	0.468 a
		Yes	245	94.20%	169	94.90%	76	92.70%	
	Imitation	No	5	1.90%	3	1.70%	2	2.40%	0.681 a
		Yes	255	98.10%	175	98.30%	80	97.60%	
	Communicates by pointing fingers	No	5	1.90%	5	2.80%	0	0.00%	0.125 a
		Yes	255	98.10%	173	97.20%	82	100.00%	
	Turns around when called	No	4	1.50%	3	1.70%	1	1.20%	0.777 a
		Yes	256	98.50%	175	98.30%	81	98.80%	
	Language comprehension	No	9	3.50%	7	3.90%	2	2.40%	0.540 a
		Yes	251	96.50%	171	96.10%	80	97.60%	
	Gets nervous in unfamiliar places	No	34	13.10%	21	11.80%	13	15.90%	0.367 a
		Yes	226	86.90%	157	88.20%	69	84.10%	
	Hyperactivity-like symptoms	No	175	67.30%	133	74.70%	42	51.20%	< 0.001 *a
		Yes	85	32.70%	45	25.30%	40	48.80%	
	Walking alone	No	5	1.90%	2	1.10%	3	3.70%	0.167 a
		Yes	255	98.10%	176	98.90%	79	96.30%	

*= *P* < 0.05, a = χ² test P, b = trend test P

**Table 3:** Association between toddlers’ behavior and mothers’ persistent postpartum depression (logistic regression analysis)

	Sleep duration		Difficulty falling asleep		Three meals per day		Eating slowly		Indoor sitting time		Smartphone use		Tablet use		Device use before bedtime		Hyperactivity-like symptoms	
	OR(95%CI)		OR(95%CI)		OR(95%CI)		OR(95%CI)		OR(95%CI)		OR(95%CI)		OR(95%CI)		OR(95%CI)		OR(95%CI)	
Age	0.329(0.127–0.852)	*	0.612(0.281–1.332)		0.616(0.251–1.513)		1.124(0.640–1.975)		0.809(0.468–1.398)		0.681(0.254–1.829)		0.706(0.219–2.273)		0.784(0.387–1.588)		0.913(0.506–1.648)	
Employment	3.625(1.638–8.024)	*	0.857(0.443–1.658)		1.587(0.733–3.435)		2.143(1.270–3.617)	*	0.469(0.284–0.775)	*	2.273(0.927–5.574)		2.377(0.807–6.997)		1.246(0.664–2.336)		1.114(0.652–1.905)	
Persistent postpartum depression	1.699(1.337–2.160)	*	1.412(1.133–1.760)	*	1.458(1.136–1.871)	*	1.251(1.044–1.499)	*	0.874(0.731–1.045)		1.757(1.319–2.340)	*	1.565(1.123–2.179)	*	1.316(1.068–1.623)	*	1.413(1.176–1.697)	*

*logistic regression analysis　*P* < 0.05

OR: Odds ratio, CI: Confidence interval

A logistic regression analysis was conducted with toddler behavior as the dependent variable, persistent postpartum depression as the independent variable, and age and employment as the adjusted variables

## Discussion

A novel finding of this study is that persistent maternal depression was associated with toddler behavior. This supports previous studies that found an impact on the mental health of toddler raised by mothers with persistent maternal depression (Giallo et al., 2015; Tainaka et al., 2022). Persistent maternal depression was associated with mothers’ inability to relax and raise their toddler and with potentially abusive behaviors. It was also associated with hyperactivity-like symptoms, short sleep duration, and irregular eating habits in toddlers. Taken together, these findings suggest that maternal mental status, such as behaviors that raise suspicions of abuse during child rearing or negative maternal affect, may play a mediating role, affecting both the mother and the child and contributing to the child’s problem behaviors. A complex set of factors is implicated in persistent maternal depression, and these factors are thought to have a multilayered influence.

Persistent maternal depression was associated with toddler behavior. We confirmed the association between Internet use (more than 1 h of smartphone/tablet use per day), sleep (short sleep of fewer than 8 h), diet (not consuming three meals every day), and mothers’ persistent maternal depression. Associations between children’s sleep, diet, and other lifestyle factors and maternal depression have been previously reported (Gui et al., 2022; Schultz et al., 2020; Armitage et al., 2009), and our results confirm these findings. According to World Health Organization guidelines (World Health Organization, 2019), for 2-year-olds, it is important that they get 11–14 h of sleep, also noting the need to reduce their sedentary time and limit their screen time to not more than 1 h. Further, exercise and outdoor play among toddlers contribute to good sleep. Regarding diet, a recommendation in previous studies has been that toddlers eat three main meals a day (breakfast, lunch, and dinner) (Moding & Fries, 2020). Children who skipped breakfast had poorer diet quality and lower total intake (Ramsay et al., 2018), which was associated with higher cardiovascular disease mortality, and skipping meals was associated with higher all-cause mortality (Sun et al. 2022). Less sleep, skipping meals, and prolonged Internet use are undesirable lifestyle behaviors for toddlers. As infants and toddlers are still developing, their lifestyle is often determined by their parents. This suggests that the mother’s mental state may have a negative impact on the child’s lifestyle. Therefore, persistent maternal depression may lead to undesirable lifestyle habits in toddlers.

The highest OR for toddler behavior was for smartphone use time (OR=1.757, 95% CI = 1.319–2.340). In Japan, excessive smartphone use by children has been termed “smartphone neglect”; this phenomenon has become a concern due to its potential to hinder interpersonal and communication skills development in early childhood (Chotpitayasunondh & Douglas, 2018; Corkin et al., 2021; Nergiz et al. 2020). It has also been reported that strong parental depression leads to neglect and accelerates children’s smartphone dependence (Mun & Lee, 2021). Moreover, persistent maternal depression was found to be associated with hyperactivity-like symptoms in toddlers, possibly influenced by excessive screen time, which has been reported to trigger developmental disorder-related symptoms (Heffler et al., 2020). These findings suggest that maternal depression may indirectly affect toddlers’ nature (i.e., causing hyperactivity-like symptoms) through prolonged smartphone use and its consequences.

Persistent maternal depression was associated with parenting feelings, confidence, and abuse-suspect behaviors. Previous studies have already shown that temporary maternal depression after childbirth is correlated with self-efficacy and parenting stress (Schwartz et al., 2015). In addition to these factors, the present study showed that relaxed feelings and confidence in parenting were relevant. Furthermore, parenting emotion was found to be a factor associated not only with temporary but also persistent maternal depression. Additionally, 36.9% of participants reported abuse-suspect behaviors, such as emotional harm or covering the child’s mouth, consistent with national data. While occasional emotional reactions may occur, repeated abusive behaviors require intervention. Supporting maternal mental health through family cooperation and accessible services is crucial. Continuous, seamless support from pregnancy onward can promote long-term well-being for both mothers and toddlers. In Japan, efforts are underway to provide seamless support through ongoing assistance. However, there are some omissions. For example, support is not given to those who have not expressed a need for help. Nuclear families are common, and there is often no one to turn to. Even if someone is in need, it is sometimes difficult to identify or grasp such challenges and situations. We believe that it is necessary to create a system in which mothers can ask for help without hesitation, along with the creation of a community where they can support each other.

Taken together, these findings suggest that the mother’s mental state, including behaviors that raise suspicions of abuse and negative maternal feelings during child-rearing, may play a mediating role, affecting both the mother and the toddler and contributing to the toddler’s problematic behavior. A complex set of factors may be involved in persistent maternal depression, and these factors may have a multilayered influence.

### Limitations

This study had several limitations. First, the survey used a recall-and-answer method, which may have introduced recall and social desirability biases. Second, the sampling may have been biased given that the participants were online survey respondents. The representativeness of the survey results and the potential bias were concerns due to the lack of information regarding the selection of survey participants and respondents. Third, maternal depression found in this study (based on the EPDS results) was higher than average, suggesting that the survey attracted relatively anxious mothers with infants. The reported impact of the non-face-to-face survey method and the COVID-19 pandemic on the parenting environment may have influenced these results as well (Gómez-Baya, D., Gómez-Gómez, I., Domínguez-Salas, S., Rodríguez-Domínguez, C., Motrico, Emma 2023). In particular, the COVID-19 pandemic has also been reported to be associated with the development of depressive and anxiety symptoms in mothers of infants and may have been an effect modification factor. Fourth, the follow-up response rate for the second survey was 44.6%, which was restricted by the low response rate and a small number of participants in the analysis; future studies need to examine long-term changes with large sample sizes. Additionally, as other environmental factors, the influence of fathers and other family members must be considered because they play a significant role in a child’s development. Finally, as the study was conducted in Japan, the results may not be applicable to other countries or cultures.

## Conclusion

Factors associated with persistent postpartum depression and toddler behavior, including short sleep and hyperactivity-like symptoms, were identified. In addition, maternal parenting feelings were one of the factors associated with persistent postpartum depression, suggesting that this factor may lead to undesirable behavior in toddlers. Although support for postpartum mothers is available in Japan, it would be desirable to have more personalized choices with regard to the duration and forms of support needed and for these choices to be considered on an ongoing basis. This would also lead to desirable parenting behavior.

## Data Availability

The datasets generated and analyzed during the current study are available from the corresponding author on reasonable request.
